# Review of the Inhibition of Biological Activities of Food-Related Selected Toxins by Natural Compounds

**DOI:** 10.3390/toxins5040743

**Published:** 2013-04-23

**Authors:** Mendel Friedman, Reuven Rasooly

**Affiliations:** 1 Produce Safety and Microbiology Research Unit, Agricultural Research Service, USDA, Albany, CA 94710, USA; 2 Foodborne Contaminants Research Unit, Agricultural Research Service, USDA, Albany, CA 94710, USA; E-Mail: reuven.rasooly@ars.usda.gov

**Keywords:** aflatoxin B1, ochratoxin A, fumonisin, cholera toxin, Shiga toxin, Staphylococcal enterotoxin, ricin, α-chaconine, inhibition, natural compounds, plant extracts

## Abstract

There is a need to develop food-compatible conditions to alter the structures of fungal, bacterial, and plant toxins, thus transforming toxins to nontoxic molecules. The term ‘chemical genetics’ has been used to describe this approach. This overview attempts to survey and consolidate the widely scattered literature on the inhibition by natural compounds and plant extracts of the biological (toxicological) activity of the following food-related toxins: aflatoxin B1, fumonisins, and ochratoxin A produced by fungi; cholera toxin produced by *Vibrio cholerae* bacteria; Shiga toxins produced by *E*. *coli* bacteria; staphylococcal enterotoxins produced by *Staphylococcus aureus* bacteria; ricin produced by seeds of the castor plant *Ricinus communis*; and the glycoalkaloid α-chaconine synthesized in potato tubers and leaves. The reduction of biological activity has been achieved by one or more of the following approaches: inhibition of the release of the toxin into the environment, especially food; an alteration of the structural integrity of the toxin molecules; changes in the optimum microenvironment, especially pH, for toxin activity; and protection against adverse effects of the toxins in cells, animals, and humans (chemoprevention). The results show that food-compatible and safe compounds with anti-toxin properties can be used to reduce the toxic potential of these toxins. Practical applications and research needs are suggested that may further facilitate reducing the toxic burden of the diet. Researchers are challenged to (a) apply the available methods without adversely affecting the nutritional quality, safety, and sensory attributes of animal feed and human food and (b) educate food producers and processors and the public about available approaches to mitigating the undesirable effects of natural toxins that may present in the diet.

## 1. Introduction

Numerous foodborne diseases result from ingesting foods that are contaminated with microbial and plant toxins. Naturally occurring food toxicants can adversely affect the nutritional quality and safety of foods. Because of a growing concern about relationships between diet and diseases and because of a growing need to improve the quality and safety of our food supply, research is needed to define conditions that minimize the levels of toxic compounds in foods. Thus, in order to improve food safety, there is a need for technologies to inactivate or inhibit toxins with food-compatible natural compounds and plant extracts. 

Most natural food toxicants possess specific sites that are responsible for their adverse effects in animals and humans. Therefore, modifying such sites with site-specific reagents that will change the structural integrity and thus prevent the toxins from interacting with cell receptor sites *in vivo* may make it possible to decrease their toxic potential.

In this review, we will present a brief overview of published studies on some possible approaches to reducing deleterious effects of the following toxins produced by fungi (aflatoxin B1, fumonisins, and ochratoxin A), bacteria (cholera toxin, botulinum neurotoxin, Shiga toxins, and *Staphylococcus* enterotoxin), and plants (ricin and α-chaconine).

## 2. Aflatoxin B1 (AFB1)

### 2.1. Thiol Adducts

Aflatoxin B1 (AFB1) is a pre-carcinogen that is transformed *in vivo* to an active epoxide [1]. Prior treatment with site-specific reagents should modify the molecule in a manner that will prevent formation of the epoxide and inhibit its mutagenic and carcinogenic activity. Because thiols are potent nucleophiles [2], they may competitively inhibit the interaction of the epoxide with DNA.

**Figure 1:**
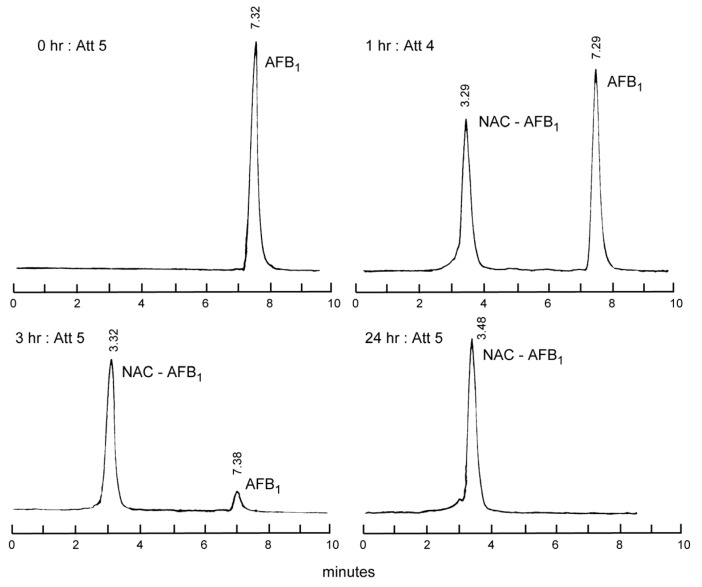
HPLC of AFB1 and AFB1-*N*-acetylcysteine (NAC) adduct. Adapted from [3,4].

**Figure 2:**
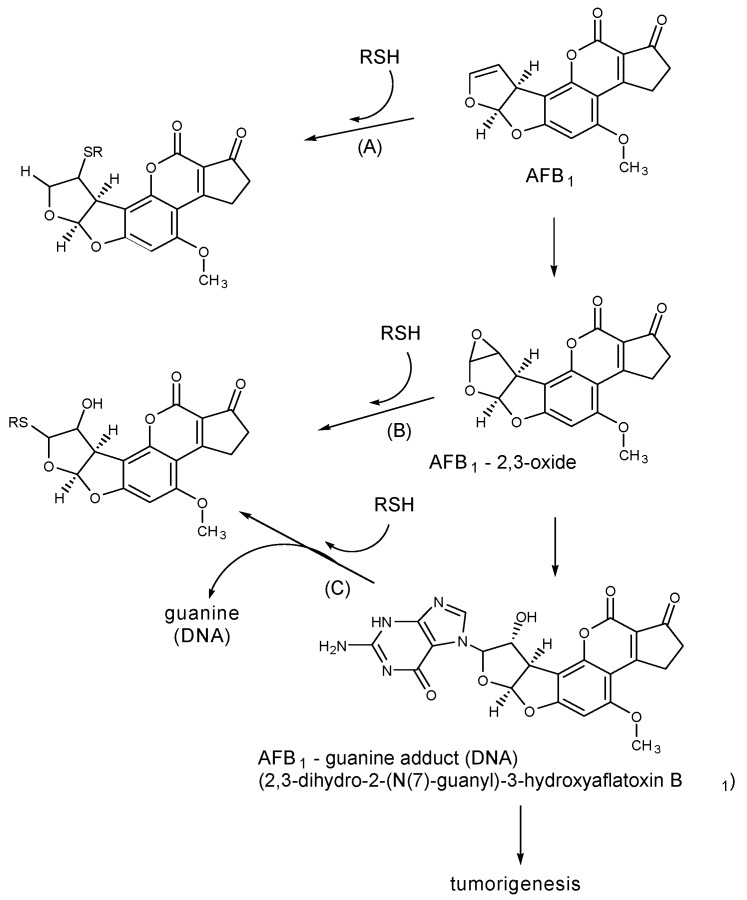
Possible pathways for the inhibition of AFB1 mutagenicity/carcinogenicity of AFB1 by thiols such as cysteine, *N*-acetylcysteine, and reduced glutathione. See text. Adapted from [3,4].

Our HPLC studies showed that exposure of AFB1 to *N*-acetyl-l-cysteine (NAC) resulted in the disappearance of the AFB1 peak and the appearance of a new peak, presumably the thiol adduct (Figure 1) [3,4]. The integrated absorbance of this peak indicated that AFB1 was converted nearly quantitatively to this single derivative. In additional experiments we found SH-containing compounds, including NAC, reduced glutathione (GSH), and *N*-2-mercaptopropionylglycine inactivated the mutagenic activity of AFB1 in the Ames *Salmonella* Typhimurium test. Surprisingly, l-cysteine was less effective. Figure 2 shows three postulated pathways for possible aflatoxin–thiol interactions. Pathway A shows the nucleophilic addition of a thiol to the 2,3-double bond of AFB1 to form an inactive thiol adduct. Pathway B depicts the interaction of a thiol with the 2,3-epoxide, which may prevent the epoxide from interacting with DNA. Pathway C shows the displacement of the AFB1–DNA (guanine) adduct, which thus prevents tumorigenesis.

Related *in vitro* and *in vivo* studies with sulfur amino acids are described by De Flora *et al.* [5,6], Shetty *et al*. [7], Guengerich *et al*. [8] and reviewed by Madrigal-Santillán *et al*. [9] and Valencia-Quintana *et al*. [10]. Cavalcante *et al*. [11] found that apple juice and cashews also exhibited anti-mutagenicity in the Ames test. 

These observations suggest that thiols may be useful for inactivating AFB1 in contaminated foods, as an antidote to treat AFB1 toxicity and for prophylaxis to prevent AFB1 poisoning.

Thiol–adduct formation also reduced the very high mutagenicity of the tetrachloroimide mutagen formed in poultry chiller water [12] and inhibited the heat-induced formation in plant foods of the presumptive carcinogen and teratogen acrylamide [13,14] as well as the antinutritional compound lysinoalanine in food exposed to heat and high pH [15]. 

### 2.2. Lysine Adducts

Several studies have reported on the formation of adducts between hydrolysis and oxidation products of AFB1 and free and protein-bound lysine residues. These include: (a) the observation that a dialdehyde derived from the *exo-*8,9-epoxide part of AFB1 reacted with both lysine and albumin to form lysine adducts [16]; (b) the finding that human AFB1 albumin adducts determined by three independent methods can be used to assess human exposure to this carcinogen [17]; and (c) a detailed description of the toxicokinetics of the serum AFB1–lysine adduct in rats [18]. These authors suggest that this biomarker has the potential to be used to relate exposure to AFB1 to human health effects.

### 2.3. Chemoprevention of AFB1-Induced Carcinogenesis in Cells

Several studies reported on the inhibition of AFB1–induced apoptosis (cell death) of cancer cells. These include the following observations:

- Rosmarinic acid, a phenolic antioxidant contained in basil, mint, and sage, prevented AFB1-induced carcinogenesis of human hepatoma HepG2 cells [19].
- Cynidin-3-*O*-β-glucopyranoside, an anthocyanin contained in blackberries, cranberries, oranges, and strawberries inhibited AFB1- and ochratoxin A-induced toxicity in HepG2 and colonic adenocarcinoma (CaCo-2) cells [20].
- Carnosic acid, a phenolic antioxidant present in the rosemary plant, exhibited a dose-dependent protective effect against apoptosis of HepG2 cells [21].
- Leontopodic acid, isolated from the aerial parts of the *Leontopodium alpinum* plant, showed chemopreventive effects against AFB1- and deoxynivalenol-induced cell damage [22].

The cited beneficial effects seem to be associated with antioxidative and/or free radical scavenging properties of the evaluated compounds.

### 2.4. Inhibition of Aflatoxicosis

Several studies describe the inhibition of aflatoxin toxicity by food compounds in different animal species. These include the following observations:

- The amino acid cysteine and methionine and yeast inhibited aflatoxicosis in rats [9,23].
- Grapefruit juice protected against AFB1–induced liver DNA damage [24].
- Garlic powder protected against AFB1–induced DNA damage in rat liver and colon [25].
- The polysaccharide mannan and yeast reduced AFB1– and ochratoxin–induced DNA damage in rats [9].
- High doses of combinations of indole-3-carbinol and crambene, compounds from cruciferous vegetables, protected against adverse effect of AFB1 [26].
- Dietary additives including cysteine, glutathione, β-carotene, fisetin, and selenium reduced aflatoxicosis in poultry [27,28].

### 2.5. Reduction of AFB1 in Food

A detailed discussion of the chemical inactivation of AFB1 in different foods is beyond the scope of this review. Reported studies include the following observations:

- Treatment with aqueous citric acid degraded 96.7% of AFB1 in maize (corn) with an initial concentration of 93 ng/g [29].
- Citric acid was more effective than lactic acid in reducing AFB1 in extrusion cooked sorghum [30].
- Extrusion cooking of contaminated peanut meal in the presence of calcium chloride, lysine, and methylamine reduced AFB1 from an initial value of 417.7 µg/kg to 66.9 µg/kg [31].
- The phenolic compounds caffeic, cinnamic, ferulic, and vanillic acids controlled aflatoxigenic fungi and the production of AFB1 and fumonisins on stored maize [32,33].
- Intermittent pumping of the volatile soybean aldehyde *trans*-2-exanal protected stored corn from *Aspergillus flavus* growth and aflatoxin contamination [34].
- The highest aflatoxin reduction (24.8%) was observed after cooking contaminated rice samples in a rice cooker, but the difference with other home-cooking methods was not statistically significant [35].

### 2.6. Practical Applications

The need to reduce the aflatoxin content of the diet is strikingly demonstrated by the observed significant reduction in the incidence of human liver cancer, especially in age groups >25 years, associated with reduced content of dietary aflatoxin [36]. The authors ascribe this beneficial effect to a shift of food consumption from moldy corn to fresh rice and improved economic status. To control fungal growth and aflatoxin and fumonisin production, drying of corn should take place soon after harvest [37]. Treatment with citric acid seems to be an effective and inexpensive method to reduce the aflatoxin content by 97%. It is not known whether the dietary ingredients mentioned above would protect humans against aflatoxicosis and liver cancer. In view, however, of the observed protection against aflatoxin-induced liver damage in albino male mice by co-administration with a black tea extract (2% infusion in water) [38], black tea may also protect humans.

These observations merit additional comment. Based on the recent *in vitro* observations by Rasooly *et al*. [39] that low levels of AFB1 stimulate growth of Vero kidney cells and high levels kill the cells, it is likely that the low residual AFB1 levels in food mentioned above would exert different and unknown biological effects *in vivo*. Further study is needed to investigate these effects in more detail.

## 3. Fumonisins

Carcinogenic [40,41] and neurotoxic [42] fumonisins, another class of fungal mycotoxins produced by *Fusarium* species and other fungal species that contaminate food, mainly grain, represent a significant hazard to the food chain [43]. For example, the consumption of fumonisin-containing maize retarded the growth of Tanzanian infants and adult celiac patients consumed higher levels of fumonisin (0.395 μg/kg) than non-celiacs (0.029 μg) [44,45]. 

Here, we present several reported studies designed to overcome fumonisin production and toxicity.

- Plant essential oils (*Cinnamomum zeylanicum, Coriandrum sativum, Melissa officinalis, Mentha piperita, Salvia officinalis,* and *Thymus vulgaris*) inhibited *Fusarium* mycotoxin production as well as fungal contamination of wheat seeds [46]. Inhibitory effects correlated with antioxidative properties of the oils. The highest inhibition of fungal growth was after 5 days of treatment and inhibition decreased after 22 days. The authors recommend the use of essential oils as natural preservatives for stored cereals.
- Fumigation of corn flour and corn kernels with allyl-, benzyl-, and phenyl isothiocyanates found in garlic resulted in a significant reduction of fumonisin content [47].
- Adsorption of the mycotoxin to a clay-based sorbent resulted in decreased bioavailability [48].
- A mycotoxin binder prevented adverse effects of fumonisin B1 in rats [49].
- Extrusion or alkaline (nixtamalisation) cooking of fumonisin-contaminated corn is an effective method to reduce potential toxicity of fumonisins [50,51].
- An ethanol extract of the plant *Aquilegia vulgaris* counteracted the oxidative stress and toxicity of fumonisins in rats [52].
- A red ginseng extract also protected rats against AFB1– and fumonisin–induced pre-cancerous lesions [53].
- Several herbal teas and extracts protected against fumonisin B1-induced cancer promotion in rat liver [54].

### Practical Applications

The cited results indicate that approaches are available to reduce the production and toxic potential of fumonisin in contaminated grain. Because, as mentioned above, plant essential oils seem to inhibit the contamination of grain by fumonisin-producing fungi and the production of fumonisins, and because a large number of essential oils and their bioactive constituents have been shown to inactivate foodborne microorganisms in laboratory media and in food [55,56,57], there is a need to optimize the anti-fumonisin potential of many of these generally-recognized-as-safe (GRAS)-listed natural compounds.

We are not aware of an approach that can be used to inhibit the toxicity of toxic weed seeds that also contaminate grain [58,59,60].

## 4. Ochratoxin A

Another fungal toxin called ochratoxin A produced by *Aspergillus* and *Penicillium* species is reported to contaminate food [61,62,63,64,65], to induce cytotoxicity in mammalian cells [66,67,68], and toxicity and carcinogenicity and nephrotoxicity in animals and humans [69,70]. The following reported observations are relevant to the theme of the present paper:

- Barberis *et al*. [71] found that food grade antioxidants and antimicrobials controlled the growth of the fungi and ochratoxin A production on peanut kernels.
- Petchkongkaew *et al.* [72] demonstrated that *Bacillus* spp. from fermented soybeans can detoxify AFB1 and ochratoxin A.
- Virgili *et al*. [73] found that native yeast controls the production of ochratoxin production in dry cured ham.
- Kapetanokou *et al*. [74] observed similar results in beverages.

The suggested approaches to reduce the toxic potential of aflatoxin and fumonisin are also expected to be effective against ochratoxin.

## 5. Botulinum Neurotoxins

Bacteria of the genus *Clostridium* produce one tetanus neurotoxin (TeNT) and seven different botulinum neurotoxins (BoNT/A,_/B,_/C,_/D,_/E,_/F, and /G) that cause the flaccid paralysis of botulism [75]. These neurotoxins have a similar four-domain structure but differ in both antigenic properties and interactions with intracellular targets. Only the L chain, the N-terminal domain of 50 kDa, enters the cytosol, where it cleaves the synaptosomal (SNAP-25) protein and blocks neurotransmitter (acetylcholine) release, causing peripheral neuromuscular blockade and flaccid paralysis in humans. Botulinum neurotoxin is highly toxic to humans. Serotype A (BoNT/A) is the most potent of several serotypes with an LD_50_ of 0.8 µg for a human weighing 70 kg [76]. Medical treatment for botulism is a major challenge [77,78].

Although rare, outbreaks of foodborne botulism are reported to occur worldwide. In the United States, Juliao *et al*. [79] reported that a commercially produced hot dog chili sauce seems responsible for four cases of type A botulism and Date *et al*. [80] reported on three outbreaks of foodborne botulism caused by unsafe canning of vegetables. These outbreaks may be the result of survival of *Clostridium botulinum* spores during preparation of these foods. 

Different food categories are reported to be susceptible to contamination by *Clostridium botulinum* pathogens. These include baked products [81,82,83], dairy products [84], fresh mussels [85] and especially canned fruits and vegetables [86]. The following observations are relevant to the theme of the present review:

- Studies by Daifas *et al*. [87] revealed that a commercial mastic resin and its essential oil in ethanol solution inhibited the growth of proteolytic strains of *Clostridium botulinum* in media. The anti-botulinal activity was greater when the test substances were applied in the vapor state than in solution. The test substances did not, however, inhibit neurotoxin production in challenge studies with the bacteria in English-style crumpets but the authors suggest that these natural products have the potential to inhibit pathogenic bacteria in bakery products.
- A reduced level of nitrite (75 mg/kg) inhibited the toxigenesis of *Clostridium botulinum* type B in meat products [88].
- The combined treatment with chlorine and lactic acid inhibited both *E. coli* O157:H7 and *Clostridium sporogenes* in spinach packaged in modified atmospheres [89].
- The thearubigin polymeric fraction of black tea blocked the toxicity of the botulism toxin by binding (chelation) to the metalloproteinase part of the toxin [90,91,92].
- Kaempfenol, kaempferol, and quercetin glycosides isolated from black tea inhibited the neuromuscular inhibitory effects of botulinum neurotoxin A in mouse phrenic nerve–diaphragm preparations [93].
- Ethyl acetate extracts of several teas mixed with botulinum neurotoxin type A also prevented neuromuscular blockade of a mouse phrenic nerve–diaphragm preparation [94] with an order of potency of the extracts of black tea > oolong tea > roasted tea > green tea (no effect).
- Water-soluble fractions of the stinging nettle leaf extract inhibited the protease activity of botulinum neurotoxin type A but not type B [95].
- Chicoric acid isolated from the herbal plant *Echinacea* is a potent exosite inhibitor of BoNT/A with a synergistic effect when combined with an active site inhibitor [76].
- The natural compound lomofungin inhibited the BoNT serotype A light chain metalloproteinase (LC) by nonclassical inhibition kinetics [96].

**Figure 3:**
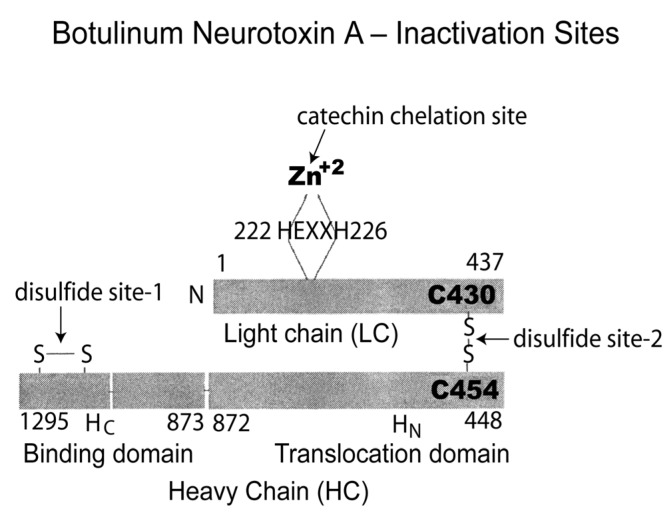
Structure of botulinum neurotoxin showing three potential sites for inactivation: zinc-containing metalloproteinase susceptible to chelation by catechin phenolic OH groups; intramolecular disulfide bond of the heavy chain (disulfide site-1); intermolecular disulfide bond linking the light and heavy chains (disulfide site-2). The disulfide bonds are susceptible to reduction and/or sulfhydryl-disulfide interchange initiated by sulfhydryl compounds such as *N*-acetyl-l-cysteine. Adapted from [102].

Šilhár *et al*. [76] state that the ability to inhibit an exosite by a small molecule requires disruption of protein–protein interactions and that natural products have the potential to act as new drugs in the treatment of botulinum neurotoxicity. The anti-toxin effect of black tea theaflavins and thearubigins and other polyphenolic compounds may result from covalent binding of the botulinum neurotoxin, possibly as illustrated in Figure 3, which depicts sites on the toxin molecule susceptible to inactivation [97].

### Practical Applications

The cited studies suggest that natural pure compounds and plant extracts added to food have the potential to help prevent botulism. Because commercial teas vary widely in their content of catechins and theaflavins [98,99], consumers have a choice of selecting teas with a high content of these anti-toxin compounds. Based on the above mentioned mechanism of inhibition of the botulinum toxin by natural polyphenolic compounds, it is likely that consumption of phenolic-rich fruits and vegetables may help protect against botulism. In addition, because there seems to be no available drug therapy, polyphenolic-rich whole foods and their bioactive compounds should also be evaluated for their medicinal properties. 

Finally, Juneja and colleagues [100,101] previously found that carvacrol (the main ingredient of oregano essential oil), oregano oil, cinnamaldehyde (the main ingredient of cinnamon oil), thymol (the main ingredient of thyme oil) and a green tea leaf extract inhibited the germination and outgrowth of the related spore-forming *Clostridium perfringens* pathogens in meat. It is not known whether these natural products would also inhibit *Clostridium botulinum* and/or the release of the neurotoxin from the pathogens in food so this aspect merits study.

## 6. Cholera Toxin (CT)

Ingestion of drinking water or cooked shellfish contaminated by the Gram-negative bacterium *Vibrio cholerae* serotypes O1 and O139 causes the potentially fatal disease cholera, characterized by profuse diarrhea [103]. Diarrhea results from the interaction of the cholera enterotoxin secreted by the bacteria with adenylate cyclase of the mucosa of the digestive tract, causing water flow from the open ion channels through osmosis. A major challenge is to overcome emerging antibiotic-resistant strains and inhibit the biological effects of the toxin. Here, we will briefly review reported studies on the inhibition of the toxin by components of the diet.

Toda *et al*. [104,105] found that tea catechins protected against experimental infection by *Vibrio cholerae* O1 bacteria and it has been shown that other polyphenolic compounds also inhibited the virulence of cholera toxin [106]. Indeed, a catechin from green tea bound to and interfered with the cell binding and internalization of cholera toxin [107]. Shimamura [108] found that SH-containing compounds such as cysteine and reduced glutathione inhibited the production of cholera toxin by *Vibrio cholerae* and that added vitamin B_12_ reversed the inhibition. These observations suggest that inhibition may result from the formation of an -S–S- bond between added thiols and toxin SH groups *via* sulfhydryl–disulfide interchange by mechanisms described in detail elsewhere for the inactivation of soybean inhibitors of digestive enzymes and other disulfide-containing protein toxins [2,109,110].

The B pentamer of the AB5 composition of CT binds to cell membranes and the A subunit acts as an enzyme after cleavage [111]. Becker *et al*. [111] examined the inhibition of galactose-rich natural substances of two AB5 enterotoxins, the heat-labile LT-1 toxin produced by *E. coli* and CT produced by *V*. *cholerae*, to bind to sites of ganglioside receptor GM1 using a specially adapted GM-1 coated microtiter-well ELISA. Compared to pure milk saccharides, skim milk powder interfered with both LT-I and CT inhibition. Fenugreek seeds were also highly active. The high inhibitory activity of binding of the toxin to the cell receptor sites by components of skim milk powder compared to numerous other galactose-containing substances evaluated may be due to the presence in skim milk of not only galactose-containing compounds but also glycopeptides and glycolipids, which may act synergistically. Related studies by Sinclair *et al*. [112] showed that sialyloligosaccharides derived from egg yolk inhibited binding of CT to GM1-OS immobilized to artificial planar lipid membranes. The authors suggest that these food-grade molecules could be used as health-promoting food additives.

Rasmussen *et al*. [103] used a high-throughput screening assay of an ~8,000 compound structurally diverse chemical library for inhibitors of *V. cholerae* motility, an activity required by the pathogens to colonize the small intestine. They discovered a group of quinazoline-2,4-diamino analogs that completely suppressed motility. The assay merits use to screen for the inhibition of motility by natural compounds. These authors use the term ‘chemical genetics’ to describe how small molecules can change the way protein toxins behave in real time directly rather indirectly by manipulating their genes.

Chaterjee *et al*. [113] examined whether red chili (*Capsicum annuum*), which contains capsaicin and other bioactive compounds, can suppress CT production in *V. cholerae*. They found that a methanol extract of the peppers and capsaicin strongly inhibited the CT production of various serogroups. The authors describe repression of transcription of virulence genes associated with the inhibition. As is the case with teas mentioned earlier, consumers have a choice of selecting peppers with a high content of capsaicin and other pungent pepper compounds [114,115].

Yamasaki *et al*. [116] note that although extracts from plants such as ‘apple’, ‘daio’, ‘elephant garlic, ‘green tea’, ‘guazuma’, and ‘hop’ have been shown to inhibit bacterial growth of *V. cholerae*, inhibiting bacterial growth may impose selective pressure facilitating development of resistant strains. They suggest that based on the above-mentioned results, a regular intake of chili peppers or other spices could prophylactically and/or therapeutically protect against cholera.

Velázquez *et al*. [117] tested in a rat model for anti-secretory activity of (-)-epicatechin, isolated from the *Chianthodendron pentadactylon* plant used in Mexican traditional medicine. The inhibitory effect of the catechin on CT was higher (56.9% inhibition) than on the *E. coli* toxin (24.1% inhibition). Computational molecular docking showed that the epicatechin interacted with four amino acid residues (Asn 103, Phe 31, Phe 223, and The 78) of the catalytic site of the toxin. The authors concluded that these studies support the use of the plant to treat diarrhea.

Pigmented rice bran inactivated multiple pathogens including *Vibrio cholerae* isolated from patients suffering from diarrhea [118,119]. It is not known whether bioactive rice brans can also inactivate cholera and other toxins.

### Practical Applications

The cited evidence suggests that natural substances are potential prophylactic and/or therapeutic agents that can be used to protect animals and humans against water and foodborne CT-mediated disease. Specifically, galactose-rich natural compounds, skim milk, fenugreek seeds, chili capsaicins, and (-)-epicatechin from a Mexican medicinal plant seem to be promising candidates to inhibit the toxicity of CT. It is not known whether any of these compounds will be effective against cholera in humans. In addition, preclinical and safety evaluation of a multivalent oral vaccine shows promise for further testing in humans [120].

## 7. Shiga/Shiga-like Toxins

**Figure 4:**
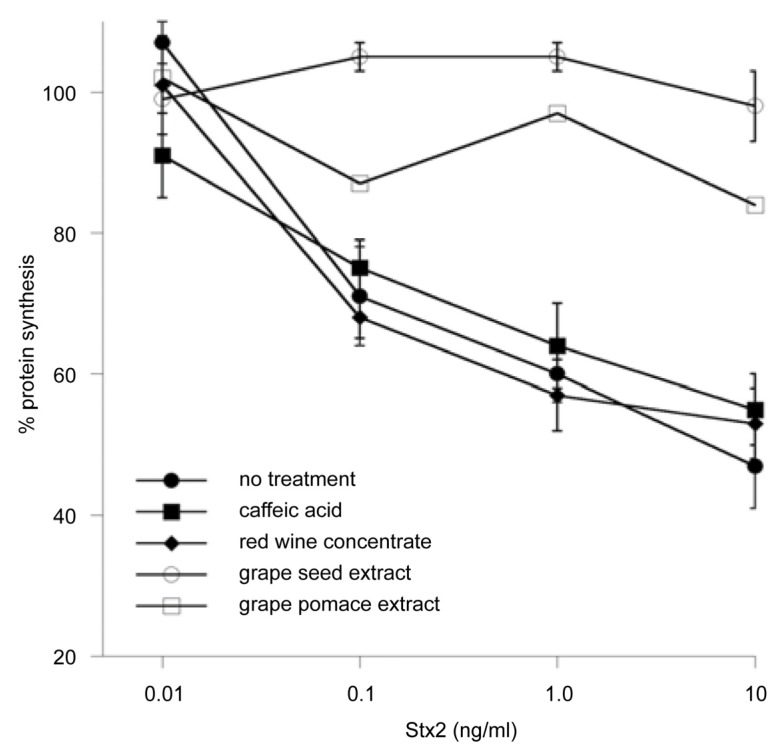
Effect of plant compounds on protein synthesis levels in Stx-treated Vero-d2EGFP cells. Protein synthesis was measured in Vero-d2EGFP cells after a 2-hour co-incubation with plant polyphenolic compounds and Stx2. Cells were co-incubated with no plant compound, 1 mg caffeic acid/mL, 1 mg red wine concentrate/mL, 0.5 mg grape pomace extract/mL, or 0.1 mg grape seed extract/mL. Adapted from [129].
- Rasooly *et al*. [130] discovered that freshly prepared juice from locally purchased Red Delicious apples, but not fresh juice from Golden Delicious apples, inactivated the biological activity of Stx2. However, both Golden Delicious juice and water with added 0.3% polyphenol-rich grape pomace, a byproduct of wine production, also inactivated the Shiga toxin. Additional studies with immunomagnetic beads with specific antibodies against the toxin revealed that only part of the added Stx2 in apple juice appears to be irreversibly bound to apple juice and grape pomace constituents. The authors suggest that food-compatible and safe anti-toxin compounds can be used to inactivate Shiga toxins in apple juice and possibly also in other liquid and solid foods. It would also be of interest to find out whether apple skin, olive, and oregano leaf bactericidal powders [131] would also inhibit Stx2.
- Different grain fractions from pea (*Pisum sativum*) and faba bean (*Vincia faba*) inhibited adhesion of enterotoxigenic *E. coli* cells (ETEC) expressing adhesion and heat-labile LT toxins [132]. Because adhesion is involved in colonization of the host by the pathogens, the authors suggest that some of the fractions have the potential to protect pigs against pathogen-induced diarrhea.
- The probiotic bacteria *Lactobacillus plantarum* isolated from a fermented milk beverage called Kefir protected Vero cells against the cytotoxicity of Stx2 present in supernatants of *E. coli* O157:H7 bacteria [133].
- A variety of probiotic bacteria, especially *Lactobacilli*, inhibited the growth *E*. *coli* strains. Whether these *in vitro* results can be confirmed *in vivo* merits study [134].

Shiga toxin is produced by *Shigella*, and the structurally similar Shiga-like toxins are produced by enterohemorrhagic strains of *E. coli* (EHEC), such as O157:H7. EHEC are pathogens of major importance for food safety, causing foodborne illnesses, ranging from mild diarrhea to a life-threatening complication known as hemolytic uremic syndrome (HUS). The bacteria produce a family of related toxins that comprise two major groups, verocytotoxin 1 (Stx1) and verocytotoxin 2 (Stx2). Stx2 is reportedly several orders of magnitude more toxic than Stx1. Stx2 is relatively heat stable and is not inactivated by pasteurization [121]. In an important *in vivo* study, Rasooly *et al*. [122] found for the first time that orally ingested Stx2, previously thought to be only dangerous when administered enterically, caused histopathological changes in kidney, spleen, and thymus, and mortality in mice. The question arises as to whether adverse effects associated with exposure to Shiga toxin-producing *E. coli* strains are caused just by the bacteria or by ingested preformed toxin as well. The following observations are relevant to the theme of this review:

- Intraperitoneal administration of 1 mg of the green tea catechin epigallocatechin gallate (ECGC) to BALB/c mice completely inhibited the lethal effect of 2 ng of Stx2 [123].
- EGCG and gallocatechin gallate (GCG) also markedly inhibited the extracellular release of Stx2 toxin from *E. coli* O157:H7 [124]. The mechanism of inhibition seems to involve interference by the catechins of the transfer of periplasmic proteins through the outer membrane of the bacterial cell. The cited findings indicate that tea compounds are potent inhibitors of Stx2. An unanswered question is whether tea compounds and teas can inactivate bacterial toxins present in drinking water and in liquid and solid foods.
- The compound eugenol, which is present in many spices, inhibited verotoxin production in a concentration-dependent manner by *E. coli* O157:H7 [125].
- The food preservatives potassium sorbate, sodium benzoate, and sodium propionate reduced Shiga toxin activity in *E. coli* O157:H7 bacteria [126].
- Glycan-encapsulated gold nanoparticles inhibited Stx1 and Stx2 [127]. The authors suggest that tailored glyconanoparticles that mimic the natural display of glycans in lipid rafts could serve as potential therapeutics for the toxins. They also note that a few amino acid changes in emerging Stx2 variants can change receptor specificity.
- In an elegant review, Branson and Turnbull [128] describe mechanistic aspects of the inhibition by multivalent synthetic scaffolds, which include glycopolymers, glycodendrimers, and tailored glycoclusters, that can inhibit the binding of bacterial toxins to specific glycolipids in the cell membrane. The authors conclude that weak interactions of inhibitors can be greatly enhanced through multivalency. The safety and food-compatibility of the synthetic inhibitors need to be established before the inhibitors can be added to food.
- Quiñones *et al.* [129] describe the development and application of an improved Vero-d2EGFP cell-based fluorescence assay for the detection of Stx2 and inhibitors of toxin activity. Grape seed and grape pomace extracts both provided strong cellular protection against Stx2 inhibition of protein synthesis (Figure 4). The identified anti-toxin compounds can be used to develop food-compatible conditions for toxin inactivation that will benefit microbial food safety, security, and human health.

### Practical Applications

The identified anti-toxin compounds can be used to develop food-compatible conditions for the inactivation of Shiga toxins in food, animals, and humans that will benefit microbial food safety, security, and human health. In addition, other natural products and plant extracts have been shown to inactivate Shiga toxin-producing bacteria [135,136]. There is a need to determine whether these and related natural products inhibit the release of Shiga toxins and whether any released toxin is susceptible to concurrent inactivation in food, in the digestive tract, and after absorption into the circulation.

## 8. Staphylococcus Enterotoxins

*Staphylococcus aureus* is a major bacterial pathogen that causes clinical infection and foodborne illness, as reviewed in Rasooly and Friedman [137]. This bacterium produces a group of 21 known enterotoxins (SEs) that have two separate biological activities: they cause gastroenteritis in the gastrointestinal tract and act as a superantigen on the immune system. Functional enterotoxins bind to the alpha-helical regions of the major histocompatibility complex (MHC) class II molecules outside the peptide-binding groove of the antigen presenting cells (APCs), and also to the variable region (Vß) on T-cell receptors. The toxin then forms a bridge between T cells and APC. This event then initiates the proliferation of a large number (~20%) of T cells that induce the release of cytokines. At high concentrations, cytokines are involved in the causes of certain human and animal diseases such as atopic dermatitis and rheumatoid arthritis in humans and mastitis in dairy cows [137]. We will now briefly mentioned reported studies designed to overcome the toxicity of the SEs, especially the virulent staphylococcal enterotoxin A (SEA), a single-chain protein that consists of 233 amino acid residues and has a molecular weight of 27,078 Da.

**Figure 5:**
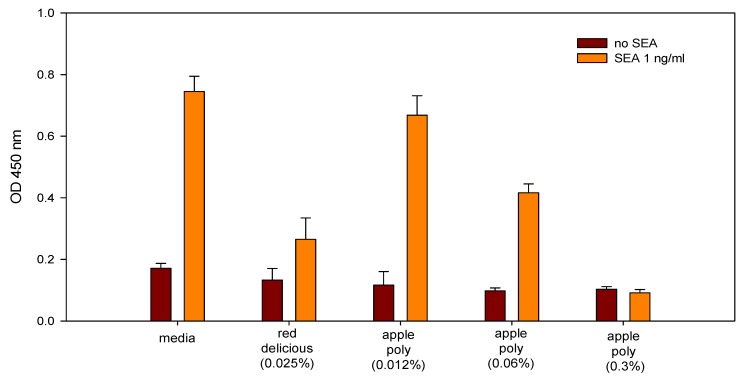
Comparison of inhibition of SEA by Red Delicious apple juice and apple polyphenols (Apple Poly). Splenocytes and SEA (1 ng/mL) were incubated for 48 h with Red Delicious juice or decreasing concentrations (0.3%, 0.06% and 0.012% w/v in PBS) of Apple Poly. The level of newly synthesized DNA was then determined, by measuring optical density at 450 nm. Adapted from [146].

**Figure 6:**
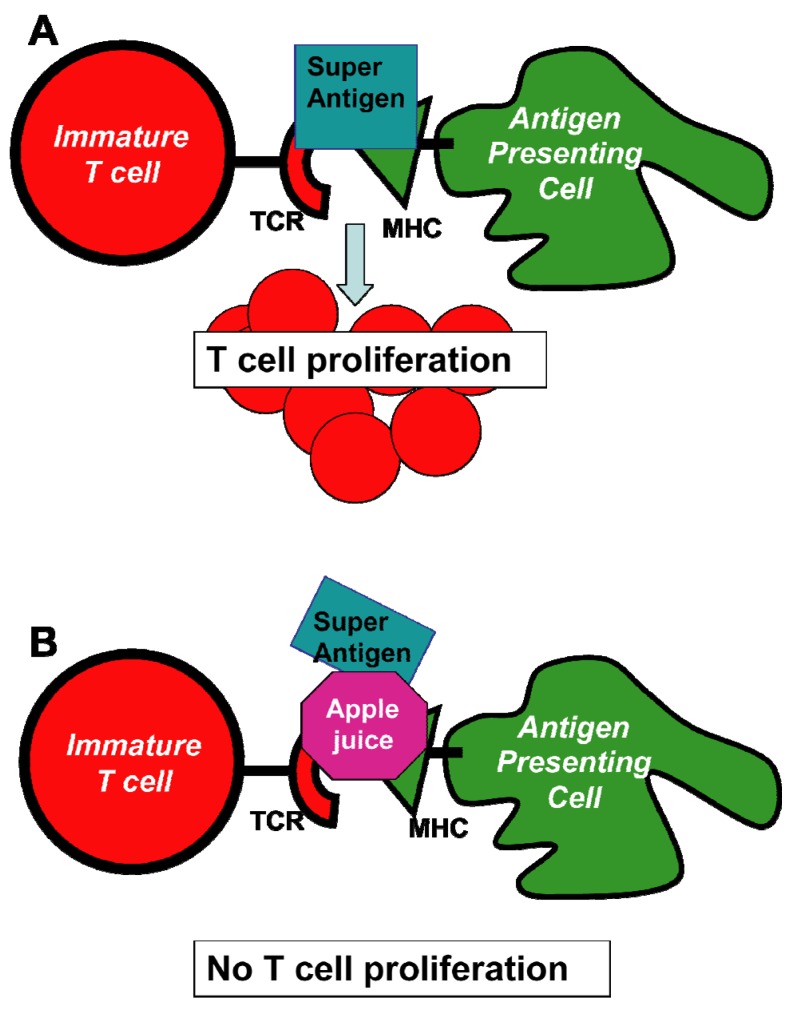
Schematic representation of cellular events that lead to the inhibition of SEA induced cell proliferation by apple juice. The individual steps in this scheme involve (**A**) the formation of a bridge between antigen presenting cells (APC) and T cells that results in the induction of T-cell proliferation; and (**B**) the inhibition of T-cell proliferation by added pure apple juice that disrupt the connection between APC and T cells. The net beneficial result of these events is the prevention of release and the consequent adverse effects induced by cytokines. Abbreviations: MHC, major histocompatibility complex; TCR, T-cell receptor. Adapted from [146].
- A dilution series of the olive compound 4-hydroxytyrosol and a commercial olive powder containing approximately 6% 4-hydroxytyrosol and 6% of other phenolic compounds inactivated the pathogen [147]. Two independent assays, (5-bromo-2-deoxyuridine (BrdU) incorporation into newly synthesized DNA, and glycyl-phenylalanyl-aminofluorocoumarin proteolysis) showed that the olive compound also inactivated the biological activity of SEA at concentrations that were not toxic to spleen cells used in the assay. Efforts to determine the inhibition of the toxin by the olive powder were not successful because the powder was cytotoxic to the spleen cells at concentrations that are effective against the bacteria. The results (Figure 7) show that the olive compound can be used to inactivate both the bacteria and the toxin produced by the bacteria and that the use of cell assays to determine inhibition can only be done with concentrations of the inhibitor that are not toxic to cells.

- Intraperitoneal administration of a green tea extract and of the tea catechin ECGC to BALB/c mice bound to and inhibited the staphylococcal enterotoxin B (SEB) [138]. The inhibition of the heat-resistant enterotoxin was both dose and time dependent. ECGC also inhibited Staphylococcal superantigens-induced activation of T cells both *in vitro* and *in vivo*. Because these antigens aggravate atopic dermatitis, the authors suggest that catechins may be useful in the treatment of this human disease.
- Ether extracts of the herb *Helichrysum italicum* inhibited the production of enterotoxins (A–D) by *S*. *aureus* strains in culture media, suggesting that the extract interfered with the production of the enterotoxins [139].
- *Lactobacillus* starter cultures inhibited both growth of *S. aureus* and enterotoxin production in sausages during fermentation [140]. The authors suggest that intestinal *Lactobacillus* strains could be used as a starter culture to produced microbiologically safe meat products.
- Microbial growth and SEA production rates of *S*. *aureus* in the presence of undissociated lactic acid can be used as indicators of bacterial growth and SEA formation during initial stages of cheese production [141].
- The sour-milk beverage Kefir with added alimentary fiber inhibited pathogenic properties of *S*. *aureus* in humans [142]. The growth inhibition of *S*. *aureus* by lactic acid produced from starter culture may be the cause of growth inhibition of the pathogen in pasteurized milk and cheese [143].
- An ethanol extract from the bulb of the *Eleutherine americana* plant inhibited both *S*. *aureus* strains and enterotoxin A–D production in broth and cooked pork [144]. The extract at 2 mg/mL delayed production of toxins A and C for 8 and 4 h, respectively, whereas toxin B was not detected in the pork after 48 h. The authors suggest that the ability of the extract to inhibit lipase and protease enzymes and to delay enterotoxin production in food indicates that it could be a novel additive against *S. aureus* in food.
- The 12-carbon fatty acid monoether dodecylglycerol (DDG) was more effective than glycerol monolaurate (GML) in inhibiting *S. aureus* growth *in vitro* [145]. By contrast, GML was more effective than DDG in reducing mortality, suppressing TNF-α, *S. aureus* growth and exotoxin production, and mortality in a rabbit model. The authors suggest that GML has the potential to be an effective anti-staphylococcal topical anti-infective candidate.
- Dilutions of freshly prepared apple juices and a commercial apple polyphenol preparation (Apple Poly^®^) inhibited the biological activity of SEA in a spleen cell assay (Figure 5) [146]. Studies with antibody-coated immunomagnetic beads bearing specific antibodies against the toxin showed that SEA added to apple juice seems to be largely bound to the juice constituents. Figure 6 depicts a possible mechanistic scheme for the inhibition.

**Figure 7:**
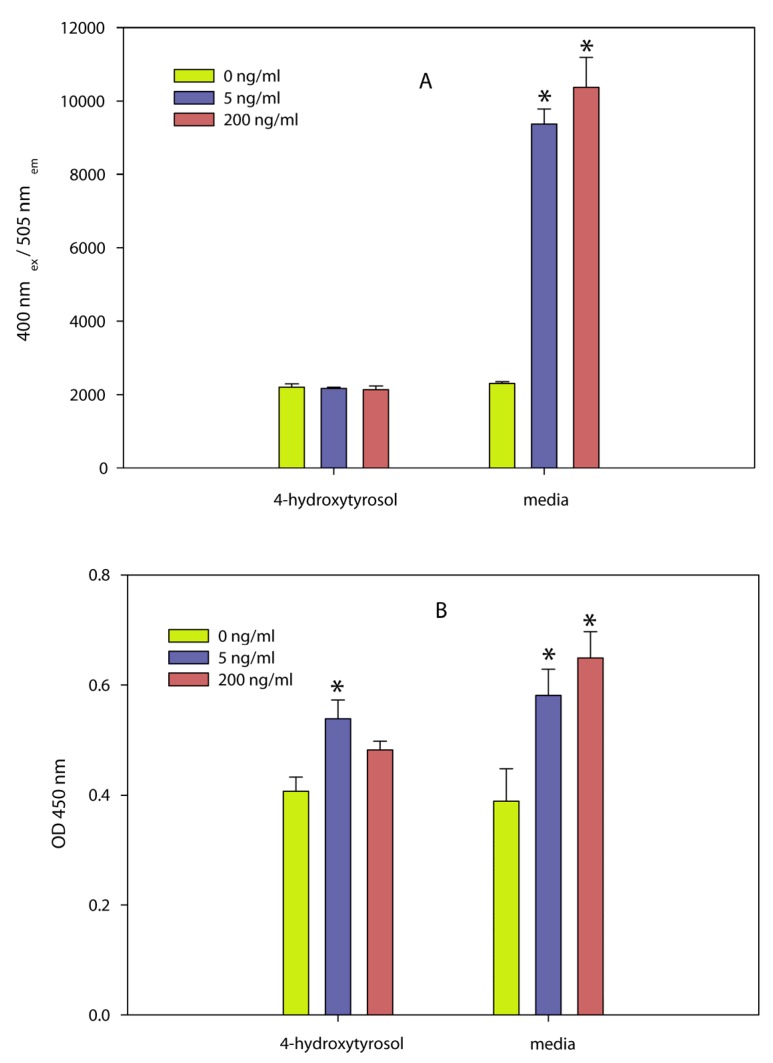
Effect of 4-hydroxytyrosol on splenocyte proliferation determined by two independent methods. Different concentrations of the toxin (0, 5, and 200 ng/mL) were exposed to 4-hydroxytyrosol or the control (media) and were then incubated for 48 h with splenocyte cells followed by determining (**A**) GF-AFC cleavage by live cell protease (a measure of cellular activity) or (**B**) BrdU incorporation into newly synthesized DNA (a measure of cellular proliferation). Conditions: (**A**) GF-AFC substrate in intact cells is cleaved by live cell protease releasing the fluorescent AFC, which is quantified at an excitation wavelength of 355 nm and an emission wavelength of 523 nm. (**B**) BrdU-labeled DNA was determined spectrophotometrically at absorbances of 620 nm and 450 nm. Both assays show that 4-hydroxytyrosol inhibited the biological activity of SEA. Adapted from [147].
- The Chinese herbal extract anisodamine inhibited the *S*. *aureus* toxin in human blood mononuclear cells [148].
- Hemoglobin inhibits the production of *S. aureus* exotoxins in a cell assay [149].
- Several phenolic antioxidants showed antimicrobial activity against several *S*. *aureus* strains [150] .
- Human monoclonal antibodies against SEB possess high affinity and toxin neutralization qualities essential for any therapeutic agent [151] .
- Several synthetic peptides inhibited the emetic and superantigenic activities of SEA in house musk shrews [152].
- Apple and olive powders and oregano leaves exhibited exceptionally high activity at nanogram levels against *S. aureus* [131].

### Practical Applications

The experimental findings suggest that apple, olive, and tea antioxidant and antimicrobial compounds and lactic acid can neutralize the biological activity of SEA. Formulations containing these food ingredients merit further study to define chemopreventive effects against SEA-induced mastitis in dairy cows and atopic dermatitis and rheumatoid arthritis in humans.

## 9. Ricin

Ricin is a heterodimeric highly toxic protein produced by the seeds of the castor plant *Ricinus communis*. In the plant, ricin is translated as a single 66-kDa polypeptide chain protein that is activated intracellularly by proteolytic cleavage to form the active 32-kDa A chain containing enzymatic activity [153,154]. The B chain is essential for the toxin’s entry into the cell [155]. The A chain is linked by a disulfide bond to the 34-kDa B chain lectin, which has an affinity to bind to cell surface carbohydrates such as galactose, galactosamine, or *N*-acetylgalactosamine present in glycoproteins and glycolipids. The toxin enters the cell by endocytosis in membrane vesicles and is transported to endosomes, and then into the cytosol. After the disulfide bond is reduced, the ricin A chain inactivates ribosomes by removing the 28S ribosomal RNA in the 60S ribosomal subunit at the adenine nucleotide (A4324) near the 3’ end of the polynucleotide chain [156]. This deletion results in the failure of elongation factor-2 to bind to the ribosome and thus inhibits protein synthesis, resulting in cell death. The low lysine content of the A chain reduces its susceptibility to proteolytic degradation in the cytosol [157]. Ricin is a highly toxic protein. A single molecule of ricin reaching the cytosol can kill that cell as a result of inhibition of protein synthesis [153]. 

**Figure 8:**
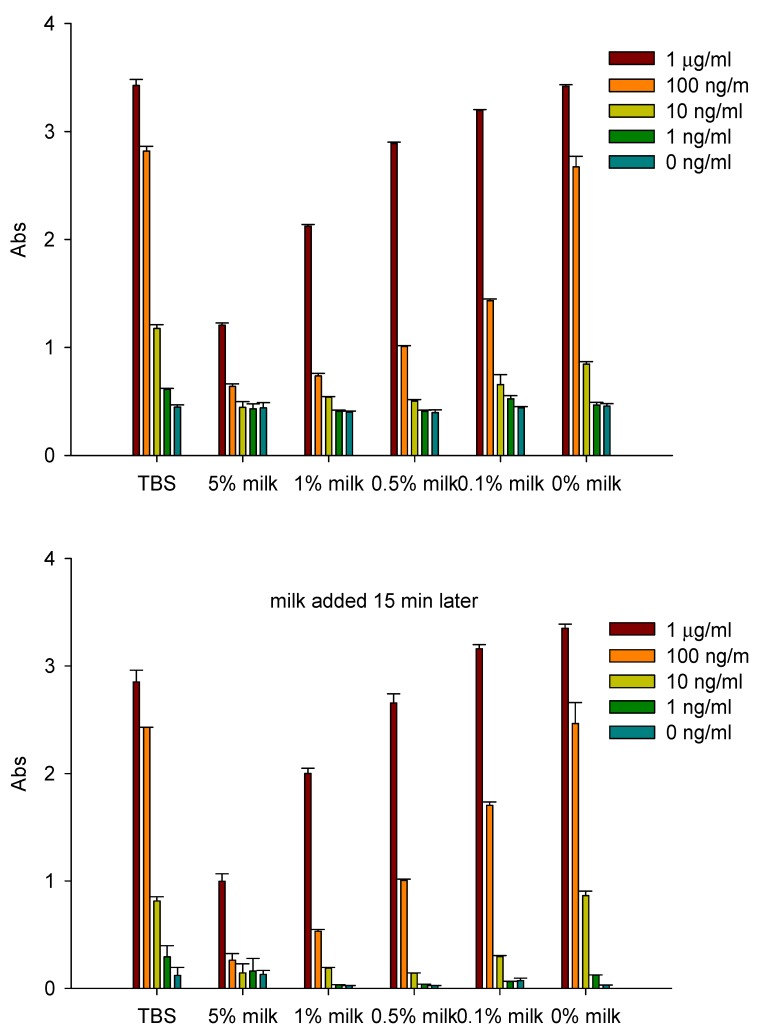
ELISA method demonstrating that milk competitively inhibits in a concentration-dependent manner attachment of ricin to asialofetuin type II coated plates (reduces the number of toxin molecules on the plate). (**A**) Upper plot: time 0; (**B**) Lower plot: after 15 min. Absorbance is read at 450 nm. Adapted from [158].

A search of the literature failed to reveal any reports of natural compounds that can inhibit the biological activity of ricin, except for the recent report by Rasooly *et al*. [158] who showed by three independent assays that components of reconstituted powdered milk have a high binding affinity to ricin. Milk can competitively bind to and reduce the amount of toxin available to asialofetuin type II, which is used as a model to study the binding of ricin to galactose cell-surface receptors. An activity assay by immuno-PCR showed that milk can competitively bind to 1 ng/mL of ricin, reducing the amount of toxin uptake by the cells and thus inhibit ricin’s biological activity (Figure 8). The inhibitory effect of milk on ricin activity in Vero cells was at the same level as by anti-ricin antibodies. By contrast, milk did not inhibit the activity at higher ricin concentrations or that of another ribosome-inactivating protein, Stx2 produced by pathogenic *E. coli* O157:H7 (see above). Unlike ricin, which is internalized into the cells via a galactose-binding site, Stx2 is internalized through the cell-surface receptor glycolipid globotriaosylceramides Gb3 and Gb4. It seems that ricin toxicity may possibly be reduced by a widely consumed natural liquid food and/or by some of its components. 

Related studies showed that (a) a sensitive *in vitro* assay can be used to detect levels as low as 200 pg/mL of biologically active ricin in food [159]; (b) a virtual screening program of 50,000 compounds enabled the discovery of new classes of ricin toxin inhibitors [160]; and (c) intra-tumoral injection of a ricin-loaded hydrogel may be useful for interstitial chemotherapy in pancreatic cancer [161]. 

### Practical Applications

The oil extracted from castor beans has been used as a lubricant, as a component of plastics, as a fungicide, and in the synthesis of biodiesel fuels. By contrast, the protein-rich byproduct, called castor bean cake or castor bean mash, that remains after cold-press extraction of castor oil cannot be used as an animal feed because it contains ricin and allergenic (2S albumin) proteins [162,163,164]. Fernandes *et al*. [162] found that solid-state fermentation of the cake with *Aspergillus niger* eliminated all ricin after 24 h. In addition, treatment of the cake with calcium hydroxide or calcium oxide completely eliminated both the ricin toxicity and albumin allergenicity. Animal feeding studies of the treated castor cake are needed to confirm the safety of the detoxified product. 

In view of the high affinity of milk compounds for ricin mentioned earlier, it would also be of interest to determine whether individual milk compounds, skim milk, or fermented milk products (e.g., Kefir or yogurt) can neutralize ricin in castor bean cake.

## 10. α-Chaconine

The potato glycoalkaloids α-chaconine and α-solanine act as natural defenses against insects and other pests, reviewed in [165]. In some potato varieties, the concentrations of these compounds can be high. High levels may be toxic to humans as well as insects. As part of a program of improvement in the safety of potatoes using molecular plant genetics and parallel food safety evaluation, we evaluated the effect of several potato glycoalkaloids and aglycones in the frog embryo teratogenesis assay–*Xenopus* (FETAX) [166]. α-Chaconine was found to be teratogenic and more embryotoxic than α-solanine, in terms of the median lethal concentration (LC50) after 96 hr of exposure, the concentration inducing gross terata in 50% of the surviving frog embryos (96-hr EC50, malformation), and the minimum concentration needed to inhibit the growth of the embryos. Since these two compounds differ only in the nature of the carbohydrate side chain attached to the 3-OH group of solanidine, the side chain appears to be an important factor in governing teratogenicity. We also found that mixtures of α-chaconine and α-solanine caused synergistic malformations and mortality and that the aglycones demissidine, solanidine, and solasodine without a carbohydrate side chain were less toxic than the glycosides. The FETAX can be used for: (a) predicting the teratogenic potential of Solanaceae alkaloids, glycoalkaloids and related natural products; and (b) facilitating experimental approaches to suppress plant genes and enzymes that control the biosynthesis of the most toxic compounds.

In related studies, we discovered that folic acid, the folic acid analog methotrexate, glucose-6-phosphate, and oxidized nicotine adenine dinucleotide (NADP) protected the frog embryos against chaconine-induced malformations (severe anencephaly in the brains and less severe malformations in the other organs [167,168,169]. 

### Practical Applications

The mentioned compounds have the potential to protect against neural tube defects and other malformations in humans. This suggestion is reinforced by the reported observations that folic acid consumption during pregnancy seems to help placental development in pregnant women and protect against neural tube defects in newborns [170]. We do not know whether glucose-6-phosphate will exhibit similar beneficial effects. Table 1 lists all the inhibitors mentioned in the text.

**Table 1:** Inhibitory effects of natural compounds and plant extracts against fungal, bacterial, and plant toxins.

Toxin	Adverse effects	Inhibitors
**Fungal**		
Aflatoxin B1	mutagen; carcinogen	apple juice, caffeic, carnosic, cinnamic, citric, ferulic, lactic, leontopodic, rosmarinic, and vanillic acids, crambene, cysteine, cyanidinglucopyranoside, extrusion cooking, fisetin, garlic powder, glutathione, grapefruit juice, lactic acid, leontopodic acid, *N*-acetylcysteine, rosmarinic acid, yeast
Fumonisins	carcinogen, neurotoxin	clay-based sorbent, essential oils, ginseng, herbal teas, isothiocyanates, *Aquilegia* extract
Ochratoxin A	cytotoxin, nephrotoxin	antioxidants, *Bacilli*, yeast
**Bacterial**		
Botulinum neurotoxin	neurotoxin, flaccid paralysis, botulism	chicoric acid, lactic acid, lomofungin, mastic essential oil, mastic resin, theaflavin, thearubigin, kaempferol, quercetin, teas, stinging nettle leaf extract
Cholera toxin	cholera disease, diarrhea	capsaicin, catechins, cysteine, epicatechin, glutathione, fenugreek seeds, galactose, quinazolines, rice bran, sialyloligosaccharides, skim milk, chilli pepper extract
Shiga toxins	diarrhea, hemolytic uremic syndrome, kidney, spleen, and thymus necrosis	bean fractions, apple juice, epigallocatechin, eugenol, fermented milk, glycan, glycodendrides, glycopolymers, grape seed extract, grape pomace extract, *Lactobacillus*, pea fractions, probiotic bacteria, yeast
Staphylococcus enterotoxin	atopic dermatitis, gastritis, mastitis, superantigen	anisodamine, apple juice, apple extract, dodecylglycerol, *Eleutherine* extract, glycerol monolaurate, green tea, *Helichrysum* extract, hemoglobin, hydroxytyrosol, kefir, olive powder, oregano leaves, sour milk
**Plant**		
Ricin	cytotoxin	anti-ricin antibodies, reconstituted milk, ricin hydrogel
α-Chaconine	teratogen	folic acid, glucose-6-posphate, methotrexate, NADP

## 11. Conclusions

In summary, the exploration of the concept of inhibiting the toxicological potential of natural toxins produced by fungi, bacteria, and plants by multiple approaches designed to prevent them from interacting with living cells has the potential of benefitting food safety and human health. It also contributes to our understanding of basic mechanisms of toxicity at the molecular level and should lead to the discovery of new ways to treat contaminated foods and people and to the development of new prophylactic and therapeutic compounds.

To facilitate further progress, future studies need to address one or more of the following aspects of toxin inhibition:

- Determine whether natural compounds can concurrently reduce both pathogens and toxins produced by the pathogens.
- Define additive and/or synergistic effects of mixtures of natural toxin inhibitors.
- Compare efficacy of natural inhibitors against toxins in different foods, including fruit and vegetable juices, milk and cheeses, cereal grains, and meat and poultry products.
- Develop anti-toxin films and coatings to protect foods against contamination by toxins [171].
- Determine whether anti-toxin effects of natural compounds and extracts *in vitro* can be duplicated *in vivo*, especially in humans.
- Determine the biological significance of low levels of residual AFB1 and ricin, which seem to stimulate cell growth.
- Explore the use of molecular biology anti-sense RNA methods to suppress genes that govern the biosynthesis of plant and microbial toxins.
